# Past and present foodscapes of a traditional fermented milk, *mabisi*, in three Zambian regions

**DOI:** 10.1371/journal.pone.0310507

**Published:** 2024-12-31

**Authors:** Taonga Chirwa-Moonga, Elise F. Talsma, Himoonga Bernard Moonga, Bas J. Zwaan, Sijmen E. Schoustra, Wilfred Dolfsma

**Affiliations:** 1 Department of Food Science & Nutrition, School of Agricultural Sciences, University of Zambia, Lusaka, Zambia; 2 Laboratory of Genetics, Wageningen University, Wageningen, the Netherlands; 3 Division of Human Nutrition and Health, Chair Group Global Nutrition, Wageningen University, Wageningen, the Netherlands; 4 Business Management & Organization Group, Social Sciences Department, Wageningen University, Wageningen, the Netherlands; German Institute of Development and Sustainability, GERMANY

## Abstract

Food serves not only as a source of individual physical sustenance but also a central element in shaping social relationships and culture within families and communities. The concept of foodscapes has emerged as a valuable framework for understanding the intricate connections between food, the environment, and society, highlighting both the physical and cultural dimensions of food. Production and consumption practices of traditional healthy foods, such as the Zambian traditional fermented milk *mabisi*, evolve over generations, a process influenced by the foodscape they are embedded in. Foodscapes can evolve as a result of several different developments, including migration, acculturation, and urbanization. This paper aims to utilize the concept of foodscape to understand why certain groups of individuals adopt diets incorporating *mabisi* while others do not. Additionally, the study identifies drivers of healthy food consumption within the broader foodscape. Adopting a qualitative approach using semi-structured interviews, along with purposive and snowball sampling, a total of 86 participants that differ in *mabisi* production and consumption were sampled from Southern, Eastern, and Western provinces of Zambia. Using thematic analysis, we found that individuals’ dietary choices were influenced by their ethnic origins and the evolving foodscape. Traditional food production practices were replaced by modern food production techniques, shifting from the use of traditional calabashes for *mabisi* production to plastic containers. Access to healthy indigenous foods has also diminished and because of urbanization and environmental degradation, wild roots, fruits, bushmeat and vegetables were no longer as readily available as before. Understanding the foodscape of a healthy traditionally fermented food such as *mabisi*, its elements, and its evolution over time, may provide insights that would promote the consumption of healthy traditional foods.

## Introduction

The concept of foodscapes has increasingly been used to describe the relationship between foods and the context in which they are produced, processed, and consumed [1]. Food not only provides us with a source of energy and other nutritional benefits, but is also a symbolic part of every community as socially and culturally meaningful relationships are developed through the preparation and sharing of food and meals [1, 2]. People express their views and opinions about food within their environments, describing the important role of history, and personal, social, or cultural experiences that inform how they ascribe importance to food [3]. Foodscapes relate to culture, which describes customs, beliefs, morals, laws, and practices that provide individuals and communities with a feeling of belonging and purpose within a given society [4, 5]. The interface between society and culture, from which sociocultural values stem emphasizes on the fact that it is the inherent nature of humans to interact with each other but also abide by specific norms, beliefs, and patterns that they are familiar with [4]. Cultures evolve, responding to influences from outside and reflections by the individuals carrying a culture and foodscape [6, 7]. Foodscapes are, therefore, not limited to the physical interconnections observed between food and the environment but also include ideas and the meanings that individuals use to describe food within their landscapes [8].

In nutrition, the food environment is more commonly used to define all the physical, economic, political, and socio-cultural factors within the food system that shape individuals’ food choices and their impact on nutrition and health [9]. This is important as the health and well-being of individuals are influenced by the environment in which they live and the food environment plays a crucial role in delivering healthy diets to individuals’ plates [10]. While some authors equate the definition of foodscapes with that of the food environment [3], it has been argued that the environment in the foodscape is not external, but includes the perceived intricate relationships that food has with humans, the environment, and culture [10, 11]. The interactions between food and the environment constantly evolve with changes in the food environment and interactions with foreign cultures. Thus, landscapes and foodscapes, like culture, are inherently temporal–they evolve [12]. Despite evolving, certain practices within the foodscape can remain constant and these include cooking recipes or production methods within a particular ethnic group such as for the production of *mabisi*. This has been attributed to individuals’ strong connections to family history and identity [13]. Understanding the foodscape is fundamental to promoting the consumption of healthy diets. Taste preferences and food habits are inherited or developed based on one’s ethnic origins, family traditions, and culture [14, 15]. Thus, the relationship between landscape and food cannot be described in isolation of culture.

Fermented foods have been consumed for centuries as part of diets across the world. While commercialized foods such as yogurt, cheese, and kefir have been widely documented, traditional fermented foods, such as *mabisi*, a Zambian traditional fermented milk, are under-researched, thereby not recognizing their potential. *Mabisi* possesses several nutrient and probiotic benefits which when consumed as part of a healthy diet, may contribute towards alleviating malnutrition and its consequences in low and middle-income countries (LMICs) such as Zambia [16–19]. It has been shown to contain appreciable levels of lactic acid bacteria (LAB) and their metabolites, the short-chain fatty acids (SCFAs) which are known to possess health benefits such as preventing the invasion of pathogens, enhancing the immunity of the host, reducing serum cholesterol levels and the risk of hypertension and other cardiovascular diseases (CVDs), reducing the risk of cancer and type 2 diabetes, and increasing the bioavailability of nutrients such as zinc and some B vitamins in food [17, 20–26]. Additionally, *mabisi* provides a good source of protein, calcium, and zinc [17].

However, food consumption is highly dependent on its acceptability in different regions of the world or a country. In Zambia, *mabisi* is indigenous to Southern, Western, and Central Provinces where it is widely produced and consumed [27]. Urbanization and subsequent intermarriages between individuals of different tribes have increased consumption throughout the country. Nevertheless, common local knowledge indicates that individuals from some parts of Zambia do not consume *mabisi* due to taste preferences and other factors. For example, although the Eastern Province ranks third in cattle production in Zambia (after the Southern and Western Provinces), *mabisi* production and consumption is low.

*Mabisi* production and consumption can be used as an example of dietary practices passed on from generation to generation, evolving through interactions, morals, laws, and individual emotions and convictions of those to which they are passed on to. While it is known that some ethnic groups in Zambia identify themselves with *mabisi* production and consumption while others do not, the specific reasons behind this are unclear. The ethnic origins of different groups, their subsequent migration and acculturation processes, and how these influence dietary patterns are key concepts to explore [28, 29]. Thus, our paper aimed to understand the past and present foodscapes of *mabisi* production and consumption in three different ethnic groups. We also sought to delineate the drivers of consumption of healthy foods that include *mabisi* and the dietary practices that acted as contributors or barriers to the consumption of healthy foods. Understanding the foodscape through indigenous knowledge on the dietary practices and the embrace of nutritious foods such as *mabisi* can be used today to increase its consumption in non-consuming areas and in turn, contribute to health when consumed as part of a healthy diet [19, 30].

## Methods

### Study area

Zambia is divided into ten provinces with 72 different ethnic groups. Ethnic groups within provinces share similar cultural origins and practices. In this regard, livestock and crop production differ in each region. The Southern Province ranks highest in livestock production, followed by Western and Eastern Provinces, respectively. This translates into Southern and Western Provinces having the highest milk and *mabisi* production. However, this is inaccurate for Eastern Province where, despite the large numbers of cattle reared, *mabisi* production and consumption are not considered a cultural norm and therefore hard to find. We, therefore, conducted this study in Southern, Eastern, and Western provinces to understand the differences in culture and origins of *mabisi*. The districts chosen for the study were Choma (Southern Province), Sinda, Chipata, and Lundazi (Eastern Province), and Mongu (Western Province) districts. Purposive sampling was used to select the districts. Choma and Mongu districts were selected based on previous research and contacts already established there [18, 27]. Only one district per province was selected as it was known beforehand that these were typical *mabisi* producers and consumers. In Eastern Province, three districts with different major ethnic groups were chosen as we aimed to identify any variations in the consumption and production practices, if any, of *mabisi* within the province where it was known that *mabisi* production and consumption were not indigenous to them.

### Study participants and selection

To understand the past and present foodscapes of *mabisi* production and consumption in three different ethnic groups, we used purposive and snowball sampling to select the elderly who would have a wider knowledge base of our research questions. Younger respondents were also included in the study using purposive and snowball sampling to observe whether historical information was passed down to younger generations (Table 1).

**Table 1:** Socio-demographic characteristics of the study participants from three Zambian regions (n = 86).

	Southern province	Eastern province			Western province	Totals %
	*Choma*	*Chipata*	*Lundazi*	*Sinda*	*Mongu*	
	*(n = 18)*	*(n = 19)*	*(n = 18)*	*(n = 12)*	*(n = 19)*	
**Gender**	**n (%)**	**n (%)**	**n (%)**	**n (%)**	**n (%)**	
Male	13 (72)	8 (42)	6 (33)	6 (50)	13 (68)	53
Female	5 (28)	11 (58)	12 (67)	6 (50)	6 (32)	47
**Age in years**						
19–29	1 (6)	0	0	0	0	1
30–39	2 (11)	4 (21)	1 (6)	1 (8)	1 (5)	10
40–49	2 (11)	5 (26)	2 (6)	2 (17)	4 (21)	16
50–59	1 (6)	7 (37)	6 (33)	5 (42)	2 (11)	24
60+	12 (67)	3 (16)	10 (56)	4 (33)	12 (63)	48
**Education level**						
No education	1 (6)	7 (37)	1 (6)	2 (17)	2 (11)	15
Primary (Grade 7)	7 (39)	8 (37)	12 (67)	8 (67)	6 (32)	47
Secondary (O-level or A-level)	4 (22)	3 (16)	4 (22)	2 (17)	10 (53)	27
Vocational	0	0	0	0	1 (5)	1
Tertiary (diploma or degree)	6 (33)	2 (11)	1 (6)	0	0	10
**Marital status**						
Single	1 (6)	0	0	0	1 (5)	2
Married	16 (89)	15 (79)	14 (78)	9 (75)	13 (68)	78
Divorced	1 (6)	4 (21)			2 (11)	8
Widow/ widower	0	0	4 (22)	3 (25)	3 (16)	12
**Ethnic group**						
Tonga	14 (78)	0	0	0	0	16
Ila	3 (17)	0	0	0	0	3
Lozi	1 (6)	0	0	0	15 (79)	19
Tumbuka	0	3 (16)	12 (67)	0	0	17
Ngoni	0	7 (37)	1 (6)	0	0	9
Chewa	0	8 (37)	3 (17)	3 (25)	0	15
Nsenga	0	1 (5)	0	9 (75)	0	12
Bemba	0	0	1 (6)	0	0	1
Mbunda	0	0	0	0	2 (11)	2
Tokaleya	0	0	0	0	2 (11)	2
Other	0	1 (5)	1 (6)	0	0	2
**Source of income**						
Agriculture	14 (78)	13 (68)	16 (89)	12 (100)	17 (89)	84
Business	2 (11)	0	0	0	0	2
Agriculture/ Business	2 (11)	5 (26)	2 (11)	0	2 (11)	13
Agriculture/ part-time employment		1 (5)	0	0	0	1

Before traveling to each district, we contacted the district officers from the Ministry of Livestock and Fisheries in the different provinces to whom the research objectives were explained. These acted as key informants who identified individuals in their areas that would fit into our selection criteria. Once completed, our team met the participants at a central location organized by the key informants where one-on-one interviews were conducted. The study participants were also requested to identify other elderly individuals in their communities who were willing to participate in the study. During each interview, written informed consent was obtained from each participant, and the full names of those that were unable to write were written down on their behalf by the interviewer. Ethical approval was obtained from the Tropical Diseases Research Centre (TDRC) Ethics Review Committee (TRC/C02/12/2020) which recommended that final approval be obtained from the National Health Research Authority (NHRA). This approval was obtained under reference number NHRA00004/23/12/2020.

A total of 86 participants were included in the study (Table 1) and these were sampled from three Zambian regions which are dominated by the ethnolinguistic groups Tonga; Nyanja-Chewa and Tumbuka; and Lozi and Nkoya, respectively. Interviews were stopped at each location once data saturation was reached (when it was observed that continuing the interviews would not generate any more information that had already been captured).

### Data collection

Images are used to improve the understanding of concepts and information that is provided in texts [31]. To enable the reader to fully understand our findings, we utilized pictures from our archives to provide context to the text provided.

Our study followed a qualitative approach using a semi-structured interview guide. Data from qualitative studies are generated in a natural setting, in the context and culture with which participants live and are familiar [32]. We chose to use in-depth interviews which are designed to capture the personal lived experiences and perceptions of individuals [32, 33]. Participants were allowed to relive their upbringing experiences and connect this to the norms and traditions surrounding their diets within the landscape in which they were raised. The interview guide was used to ensure that all of our research questions were addressed and this also provided structure to the interviews. The interview guide included questions on the participants’ backgrounds and their *mabisi* production and consumption methods and habits. Participants were also asked questions about other foods that they perceived to be healthy, as well as those they were taught to avoid or consider taboo during their upbringing. When they asked what was considered healthy, no specific explanation was provided, but examples of healthy foods were offered to them. The interview guide was pilot-tested on three *mabisi* consuming participants from the Department of Food Science and Nutrition who had ethnic origins from the three participant regions. The interview guide was then modified accordingly based on the responses given.

Data collection in each area was conducted at different times to allow for analysis and adjustment of questions accordingly. From 16^th^ to 18^th^ December 2020, data collection in Choma, Southern Province was conducted with 18 respondents. In the Eastern Province, a total of 48 interviews were conducted in Chipata, Lundazi, and Sinda Districts. The interview guide in Eastern Province was altered during the data collection process as it was realized that some of the questions were irrelevant for non-consuming individuals. As the interviews progressed on the first day of data collection in Chipata, questions were generated from the responses given, and these were used to alter the interview guide for subsequent interviews within the province. Data collection in Eastern Province was conducted from 29^th^ March to 1^st^ April 2021. This was followed by data collection in Mongu district, Western province, where we conducted 19 interviews from 16^th^ to 17^th^ June, 2021.

As a standard, interviews were conducted in the local language and when the lead researcher was unable to converse in some of the languages locally used, the research assistant provided assistance with translation. Whenever a participant was able to understand and converse in English, the interview was conducted in English. Reflexivity was an important aspect of these interviews, as we already had preconceived knowledge about the norms and traditions of each province. The main author of this paper is Zambian and originates from the Eastern Province. She, therefore, already had preconceived knowledge of the cultural practices of this Province. Additionally, author HBM is also Zambian and originates from the Southern Province. Authors HBM and SES conducted previous *mabisi* research in Southern and Western Provinces, and therefore, also had preconceived knowledge about some norms and practices of *mabisi* production and consumption [18, 27, 30, 34]. Thus, we took care to ensure that we did not ask any leading questions during data collection, but rather, allowed the participants to provide their own opinions on the questions asked. Probing questions were asked to explore emerging issues.

### Data analysis

Recordings of each interview were translated into English and transcribed using MS Word. This was done by the research assistant who was conversant with the languages used. Each transcribed interview was checked for quality by the lead researcher to ensure that the transcription reflected the recorded interview and everything in the interview was captured.

To analyze our data, we used thematic analysis which has been described as a flexible tool used to identify and analyze themes [35]. The transcription and quality checking process was used to familiarize ourselves with the data, after which coding of the transcribed interviews was done with reflection and memo writing where appropriate. We analyzed our data deductively, deriving emerging themes from the data collected. Initial sub-themes were generated from the codes with further reflection, taking into consideration the context of the research question. The coding process involved several steps of open coding and recoding. Where codes were generated later in the coding process, interviews that were already coded were recoded to ensure that they were applied to all the interviews. This process was repeatedly examined by two coders, with some of the sub-themes reassigned or re-generated as new codes were generated or applied. The two coders discussed the code groupings at length until a consensus was reached.

Coding and data management was achieved using Atlas.ti (version 9.0.0.214, and version 22.0 ATLAS.ti Scientific Software Development GmbH, Berlin, 2021) and Microsoft Excel. To ensure credibility of the data analysis process, the main researcher consulted the co-authors and the research assistant to examine and discuss the emerging themes.

## Results

### Ethnic origins

#### Southern province

Several participants were able to trace their origins to different parts of Africa. Most Tonga participants in Southern Province believed that they were the first settlers in the area, with most being unable to trace their origins beyond the borders of Southern Province (S1 Table). This was also true for some sections in the Ila ethnic group that mentioned that they landed from God onto the land where they settled:

*Briefly explain to me about where your ethnic group/ tribe came from and how they came to settle down in this area*

*“*… .*there are many stories*. *The word Namwala came from a big flat stone on the tributaries of the Kafue river where you find a lot of footprints of human beings*, *animals*, *and snakes*, *they are there even now*. *The Ila people say that is where they landed when they came from God which is untrue*. *Those stones were curved by pigmies*…*there are so many stories*. *We are likely to have come from East Africa in the Sudanic region*. *Others were left in East Africa and others went to South Africa*. *They came round and this is why you find Tonga-speaking people in Malawi*. *We first landed in Kalomo according to written history*… .*” (Ila male*, *60+ years old*, *Choma)*.

The love for their land and their connections to their ancestors at this stone, from which the town name Namwala originated from was evident. They related their way of life as cattle herders to this place of origin. The Ila people are known to have the largest herds of cattle in Zambia, making them very wealthy. However, as quoted above, more educated participants traced their origins to East Africa where cattle and milk production are high and the indigenous people have been known to produce different types of fermented milk [36].

#### Western province

The Lozi participants from Western Province traced their origins to Congo where their queen Mbuyawamwambwa migrated from. Around 1860 when the Kololo from Lesotho attacked Barotseland where they lived, they were defeated and the Kololo ruled them for 22 years. Participants reported that the Kololo imposed their way of life and their language on the Lozi people. It is possible that the Kololo may have also imposed the practice of *mabisi* production and consumption as it has been reported that this practice is very similar to the people of South Africa where the Kololo migrated from [37, 38].

#### Eastern province

Participants from Eastern Province traced their origins to different parts of Africa; mainly Mozambique (for participants from Sinda), Malawi, and/ or South Africa (for participants from Lundazi and Chipata) (S2 Table). Both groups of participants stated that their forefathers migrated as a result of wars they were fleeing from. The changing environment that ensued as a result of war ejected them into a different landscape where they had to adapt by adopting the way of life in the places where they settled, while at the same time losing parts of their language and identities with the acculturation that occurred. Others from Lundazi mentioned that their forefathers did not have enough farming land in Malawi which prompted them to migrate into Zambia in search of farming land. There, they acquired huge farming land on which they grew their crops. This may serve as an explanation for why these individuals continue to be recognized for their substantial maize production compared to other regions in Zambia.

### Foodscapes of *mabisi* production and consumption

#### *Mabisi* consumers

*Mabisi* production and consumption were most prominent in Southern and Western Provinces, with a few consumers in Eastern Province. Consumers considered it as a normal regular food, consumed daily without any specific religious or traditional significance attached to it. They ascribed its importance to the fact that it was a readily available food and was a good source of energy and satiety. *Mabisi* was considered as food that should be readily available in every household as it was commonly offered to visitors as well, who would often times appear on people’s doorsteps without any reservation. It was therefore considered good practice to have some food available to offer their guests. This was more commonly practiced by participants from Southern Province. *Mabisi* was valued not only for its enjoyable taste but also for its health benefits by all consumers. In Western Province, it was believed that their common practice of consuming *mabisi* and other foods such as the head and brains of fish made them very intelligent.

*Mabisi* consumers reported that when consumed, it would keep them satisfied throughout the day, particularly when working in the field or when children went out to herd cattle. It was also given to children as a snack that was consumed at school or at home. *Mabisi* was consumed in many different ways which included consumption on its own as a drink or thick yogurt-like mixture, or mixed with other foods such as rice, sweet potatoes, pumpkin, or leftover *nsima* (a stiff porridge often cooked using maize meal flour, or sometimes from millet, sorghum, and/ or cassava flour). It is common knowledge that *nsima* is the Zambian staple food which is consumed with sides that include vegetables and a protein such as eggs, fish, meat or legumes. When these are unavailable, consumers mix the *nsima* with *mabisi* and this is consumed as a main meal. Almost all consumers mentioned that *mabisi* was often times used as a replacement for relish in times when they did not have any and therefore was a cheaper alternative:

*" It is just taken like any other food, no special reasons for consuming it. It can take the place of relish in meals and it is even cheaper. (Tonga female, 60+ years old, Choma).*

*Origins*, *production and consumption of mabisi*. When participants from Southern and Western Provinces were asked where the practice of *mabisi* production and consumption originated from, they either stated that it was a tradition passed down through generations and they were unsure of its exact origin, or they reported that it was a result of accidental circumstances:

*Was mabisi part of the foods that they (your forefathers) ate? If yes, kindly explain how they first learnt of making and consuming mabisi?*

"I think it happened that they milked and ate what they could each day and left what remained in the vessel. The following day they found it had become a heavy solidifying liquid. They started calling each other to see what had happened and then stirred it as everyone was watching. They tasted it and found that it was nice and they shared it amongst themselves. This is how they started fermenting the milk overnight. From then, excess milk was not wasted. It all started with excess milk that was left to ferment overnight." (Tonga female, 60+ years old, Choma).

*"Some of these things were done as experiments*. *They did not even know they were doing something good*, *they just discovered that it came out well and it was nice to eat*.*" (Lozi male*, *60+ years old*, *Mongu)*

Unfortunately, most of the younger participants were unable to locate the origins of *mabisi*:

*Was mabisi part of the foods that they ate? If yes, kindly explain how they first learnt of making and consuming mabisi?*

*“Yes*, *but I do not know how it started*. *I just found it that way*.*” (Tonga male*, *30+ years old*, *Choma)*

**Fig 1:**
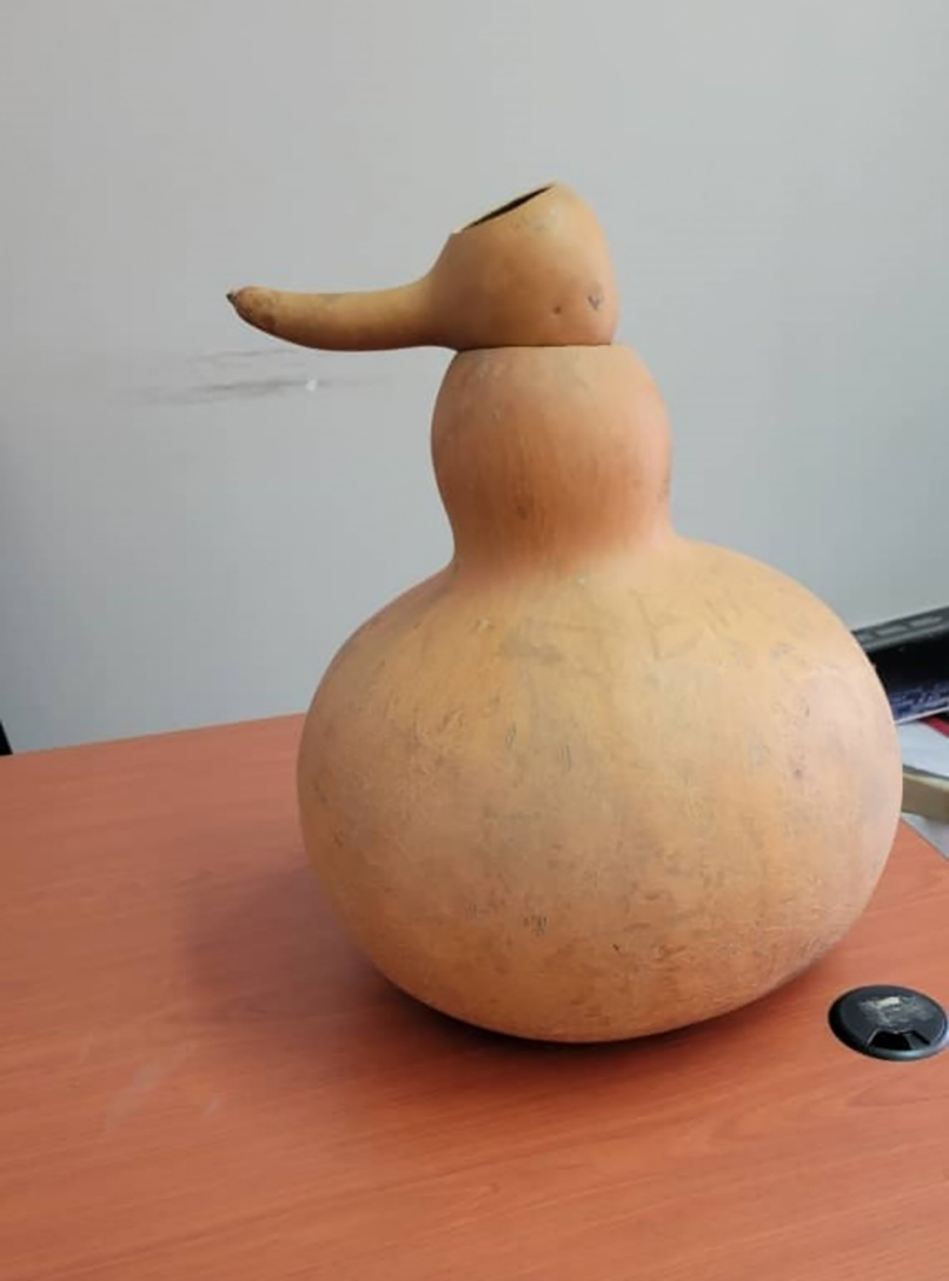
Calabash used to ferment *mabisi*.

**Fig 2:**
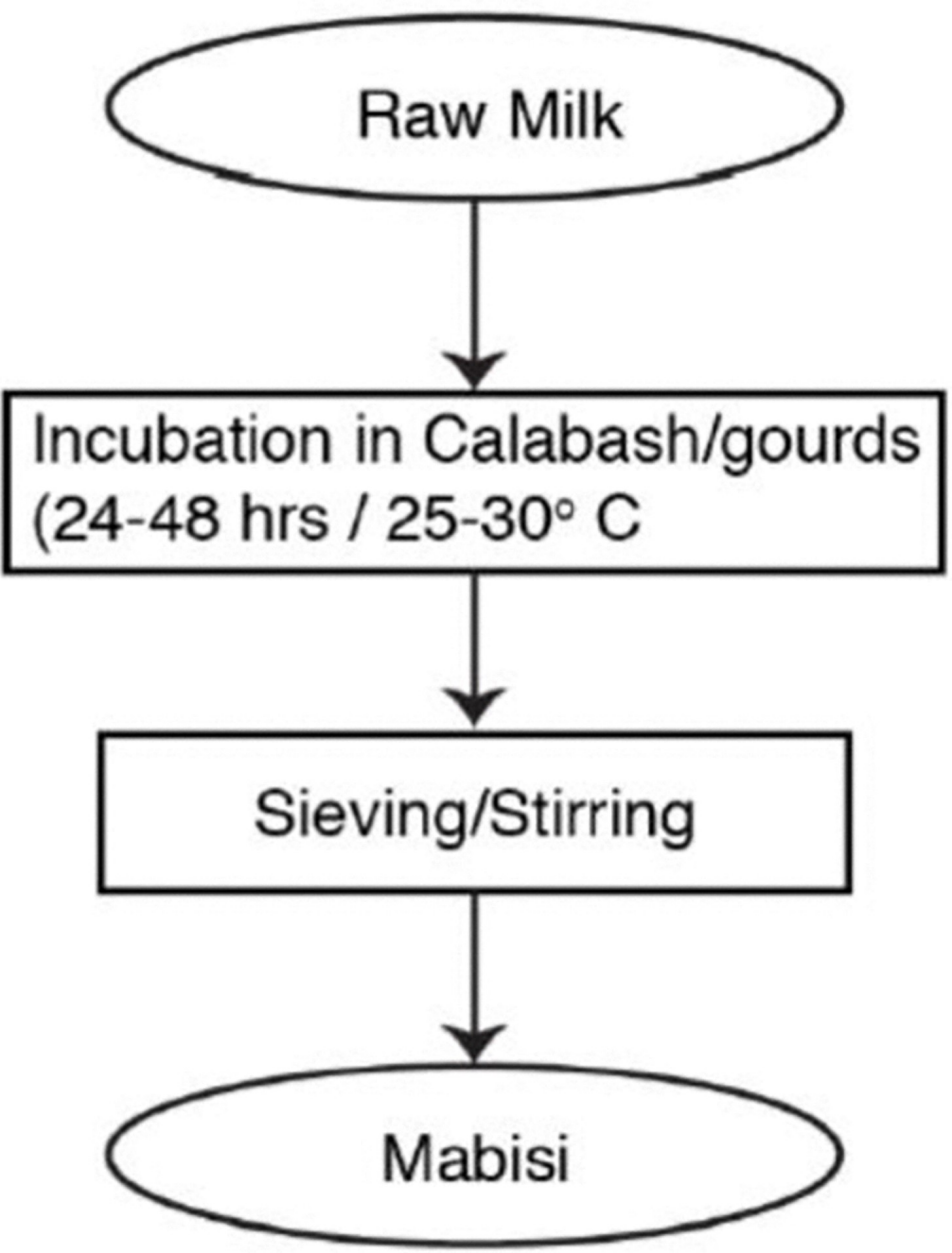
General process flow of *mabisi* production [34]. Variations exist based on location [27].

Most participants reported that their forefathers used gourds and calabashes to ferment their milk into *mabisi* (Fig 1). The methods and source of milk used to produce *mabisi* are generally the same with only slight variations depending on taste preferences and identity (Fig 2). Some participants preferred a more stiff and sour product that was kept to ferment for a longer period of time, while others preferred a more watery product that resembled drinking yogurt. Many participants ascribed meaning to the type of *mabisi* that was traditionally prepared by their ethnic group as described by [27]. For instance, when asked whether they consumed *mabisi* produced from goat milk, it was dismissed as a practice that was not part of the Lozi ethnic group but that of the Tongas:

*“No, unless the Tonga people who milk goats. Here we don’t.” (Lozi male, 40+ years old, Mongu)*

However, it was observed that goat milk was not common in any of the three subject areas and many participants responded negatively when they were asked whether they would consume it if made available. Nonetheless, a few respondents felt that goat milk was healthier than cow milk and mentioned that it produced more cream and therefore would make very good *mabisi*. This group of participants was willing to produce *mabisi* using goat milk if made available in large quantities.

The Ilas in Southern Province reported that elderly men were responsible for making *mabisi*, continuously shaking the calabash throughout the day until the *mabisi* was formed.

**Fig 3:**
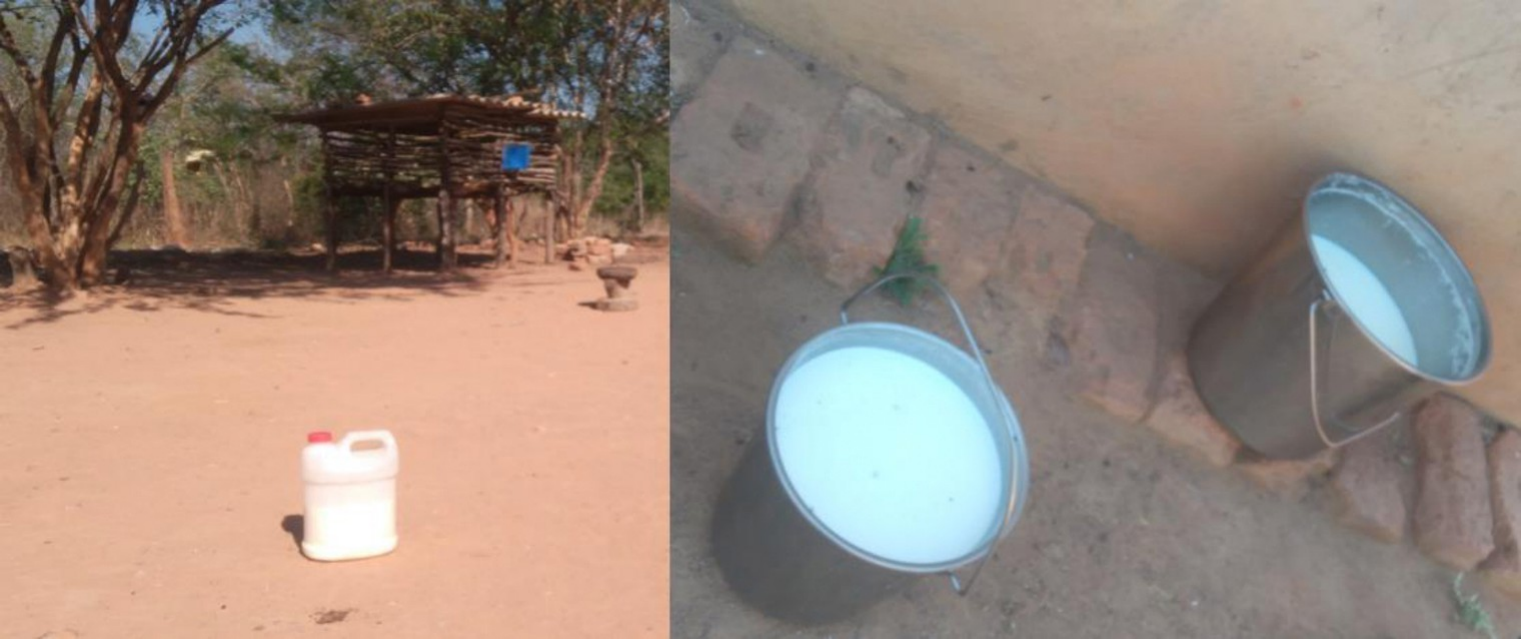
*Mabisi* produced in plastic or metal buckets.

In contrast, the Tonga women were responsible for making *mabisi*. They allowed the fresh milk to stand in a calabash or gourd overnight for 24 hours (or more) when the *mabisi* would be ready for consumption. This showed differences in the foodscape between the two ethnic groups that are found in the same province. Those from Western Province reported that while they also used calabashes to ferment their milk, they formed a hole on the side bottom of the calabash which was used to drain the whey, after which they would add fresh milk and leave it to ferment again. This process was continued for three to seven days (depending on their taste preferences). The fermentation time depended on the container and the temperatures. Many participants reported that they had discontinued the use of calabashes and gourds because they did not grow them anymore and they were no longer readily available. They had resorted to using plastic or metal buckets and containers which they reported to be more convenient (Fig 3). They also mentioned that the plastic and metal containers were cleaner than the calabashes which they felt accumulated a lot of dirt and that the calabashes were not as durable as the plastic and metal buckets:

*“I think it is because of the coming of modern technology with new equipment that can be used to ferment mabisi. Also, calabashes would break when they fell down. The cans we have do not break easily, they have a long lifespan.” (Tonga male, 19+ years old, Choma)*

They did, however, report that *mabisi* produced in calabashes was tastier and that they much preferred it over the one produced in plastic containers.

All participants were aware of *mabisi* and reported that it was consumed on its own or with other foods such as rice, *nsima* or pumpkin.

#### *Mabisi* non-consumers

In an attempt to understand why most participants in Eastern Province did not consume *mabisi*, we asked them if they or their forefathers produced and consumed *mabisi*. Their responses were mixed. Several reported that they did not consume *mabisi* and that it was not part of their culture:

*“No we never left any milk to stand overnight. In fact, this sour milk you’re talking about, I never heard of it until I was an adult….I have never had sour milk.” (Nsenga male, 50+ years old, Sinda).*

*“No because we do not see it*. *We have just heard that people from Southern province consume it but here in Eastern province we do not have it*.*” (Ngoni male*, *60+ years old*, *Chipata)*.

However, it appears that despite not being popular, *mabisi* production and consumption was practiced by some of the Eastern Province ancestors. Participants mentioned that their forefathers did not move with cattle during migration but acquired them in the areas where they settled as an adoption and embrace of the new landscape. This changed their foodscape as they were now able to produce and consume milk, cow meat and possibly *mabisi* from the herds that they reared. Based on the main author’s understanding, interviewing the participants, and reading on the subject, it is possible that the practice of *mabisi* production may have been acquired from the people they interacted with during the migration process. Participants mentioned that *mabisi c*onsumption later discontinued mainly due to the loss of animals to diseases. This reverse change in the foodscape resulted in fewer animals that were available to produce enough milk for *mabisi* production, which consequently resulted in a gradual loss of interest and taste for *mabisi* over successive generations (S1 Fig). Other participants that had a preference for *mabisi* but did not have any source of milk felt that purchasing it was a waste of money and therefore chose not to consume it at all. Participants that mentioned that their forefathers consumed *mabisi* were mostly from poor backgrounds as was evidenced by an example given below:

*Could you tell me a little about yourself, where you were born and your upbringing?*

*“……*.*we did not write on paper at the time we used to write on the floor…*.. *we lived a hard life because my father did not have any job…*..*I went up to grade 7 but for me to go to form 1 my father did not have any money to take me*. *I therefore got married but that was when my life turned for the worse*. *I did not have a peaceful life but God blessed me with children…*…*Right now I live with my husband and all we do is sell some bananas whenever they are ready for sale*.*” (Chewa female*, *60+ years old*, *Lundazi)*

In contrast, those that mentioned that their forefathers did not consume *mabisi* were from higher-income backgrounds. Leftover milk was thrown away in non-consuming households and considered to be rotten milk, while others stumbled on the product accidentally (as mentioned by those from Southern and Western Provinces below) and considered it a good product. The foodscape during this time was characterized by having excess milk that was discarded by some and used to produce *mabisi* by others. The main author of this paper derives her origins from the Eastern Province and did not grow up consuming *mabisi*. Despite the fact that she lived in a more cosmopolitan town with regular interactions with consumers, her family still did not adopt this practice. During this study, she informally asked her father and grandfather about why they did not consume it. Their answers were consistent; “We do not consume rotten milk, it is not part of our culture.” When she probed why not, her grandfather explained to her that when he was growing up, they would sell fresh milk to the Indians that lived near the village where he lived. This provided a ready source of income for other foods that they purchased.

Pride and identity also played an important role in *mabisi* consumption, with specific groups identifying themselves with a certain way of life and did not want to “corrupt” the norms and traditions they were identified by. Study participants from Eastern Province did not want to be associated with *mabisi* consumption, with most consumers reporting that the practice was only adopted when they interacted with the Tonga or Lozi ethnic groups from Southern and Western Provinces, respectively.

### Foodscapes of other healthy foods

#### The food environment

This section primarily focuses on the food environment as being part of the foodscape. Only those elements of the food environment that were reported by the participants as being important for their consumption of other healthy foods are reported here.

*Food availability and accessibility*. Participants described the importance of wild fruits and vegetables in the diets of their forefathers and in their upbringing. They consumed different types of bushmeat and wild vegetables and roots such as *mupama and kabombwe*. However, the foodscape changed over the years, with most of the bushmeat being unavailable to them as they mentioned that the animals may have been depleted, ran away due to urbanization, or stricter regulations had been placed by the government to conserve them. In response to the question about the availability of wild vegetables and roots, most participants reported that while they were still available to them, the foodscape had changed offering a wider variety of edible plants with the introduction of more cultivated vegetables, roots and tubers such as rape, cabbage, sweet potatoes and potatoes which were more desirable than the wild ones. They also mentioned that their children would not consume these foods as they were not familiar with them.

Participants further reported that with time, food availability in the villages had deteriorated as they were unable to find most of the foods that they consumed when they were younger. While participants acknowledged the changing food environment with a rise in the number of food outlets in their settlements, providing increased accessibility to processed items like cooking oil, these foods remained unavailable in their diets. This was attributed to the fact that they required money to purchase food, as opposed to the past when food was readily available in the wild without such financial demands:

*“The type of food we used to eat a long time ago has changed because there was a lot of food then, but now there is even more food but requires that you have money.” (Chewa female, 50+ years old, Lundazi).*

Farming methods and inputs also changed over the years. Crops such as rice, maize, and cassava were commonly grown in Mongu, while in Eastern Province, the commonly grown crops were millet, sorghum, maize, groundnuts, sweet potatoes, beans, and sunflower. In Southern Province, the commonly grown crops were maize, sweet potatoes, pumpkins, and millet. Despite these crops still being available, the introduction of new varieties that require fertilizers, herbicides, and other farm inputs to achieve a good yield affected their availability. Also, growing crops such as millet and sorghum was no longer desirable in some sectors as they were considered to be less profitable than growing other crops such as maize and rice:

*“Ahh sorghum is better and not millet. If you have a certain portion of land, there are a lot of losses and you get very little from it as compared to sorghum and maize. But if you grow rice on the same portion of land, you get a lot more bags.” (Lozi male, 60+ years old, Mongu).*

*Food affordability*. Farming activities form the central core and source of food in societies throughout the world. Several participants reported that they grew up in homes that reared cattle which served as a source of food or income for their households. As earlier mentioned, the entire subject population reported that their forefathers possessed large herds of cattle but these dwindled over time due to animal diseases and other factors. It is unknown where these animal diseases came from but it is clear that the changes in the landscape through these animal disease outbreaks negatively affected the health and well-being of these groups, adversely affecting their source of food and income. The significance of a healthy landscape has been emphasized by some indigenous communities by asserting, "if the land is sick, we are sick" [39]. The loss of animals was much higher in Eastern Province where it is possible that their cultural practices may have also contributed to their loss of cattle. Participants from Eastern Province reported that while they reared their cows for milk, they also slaughtered them for meat and shared it among their community members. In contrast, in Southern Province, it is unacceptable to kill cattle for meat. Instead, cattle herds have always been a symbol of wealth, and the cows were only slaughtered during festive periods, when they had bereavements, or when the cows were sick:

*“Cattle were kept as a symbol of wealth. If you did not have a lot of cattle, it was a disgracing thing. We even had traditional dances and songs to show off cattle wealth.” (Ila male, 60+ years old, Choma).*

In all three provinces, cattle were sold as a source of income to pay for their children’s school fees. Other commonly reared animals included goats (typically in Eastern Province and not Western Province), pigs (typically in Western Province), and chickens.

#### Other dietary practices

The motivation to slaughter animals for consumption was largely driven by festivities and family events. Family taboos and traditions dictated that meat could only be consumed during Christmas or funerals. When a cow fell ill, the cow would also be slaughtered for consumption. Their diets were therefore mainly comprised of chicken meat or mice (in Eastern Province), when available, rather than cow meat. Monitor lizards (*hopani*) and tortoise meat were considered delicacies in Western Province and were consumed only by men. In response to the questions asked why they were only consumed by men, it was reported that it was just because it was rare meat that was kept only for the man of the house to enjoy. The men cut the monitor lizard into pieces and boiled it for approximately six hours when it would be ready for consumption:

*“So yeah hopani is one of those, it’s a delicacy…….. and it is only prepared by men…..and you don’t cook it inside the yard. You cook it behind the yard not inside…. after some good 6 hours of cooking that’s when you remove the skins and the bones then you fry the meat. Very tasty like mincemeat, share it. You know the way we live in Western province we have the home set covered by some reed fence so that when you cook in your house nobody will know whatever you are cooking. But now since hopani is exposed outside you are forced to give other people…” (Lozi male, 60+ years old, Choma)*

More commonly consumed foods throughout our subject population included groundnuts (and groundnut powder mixed with other relishes), wild fruits, vegetables, and roots; and cereals such as maize, millet, and sorghum. Some aspects of the local foodscape have remained the same as is evidenced by the consumption of these foods which have been in these communities through generations.

**Fig 4:**
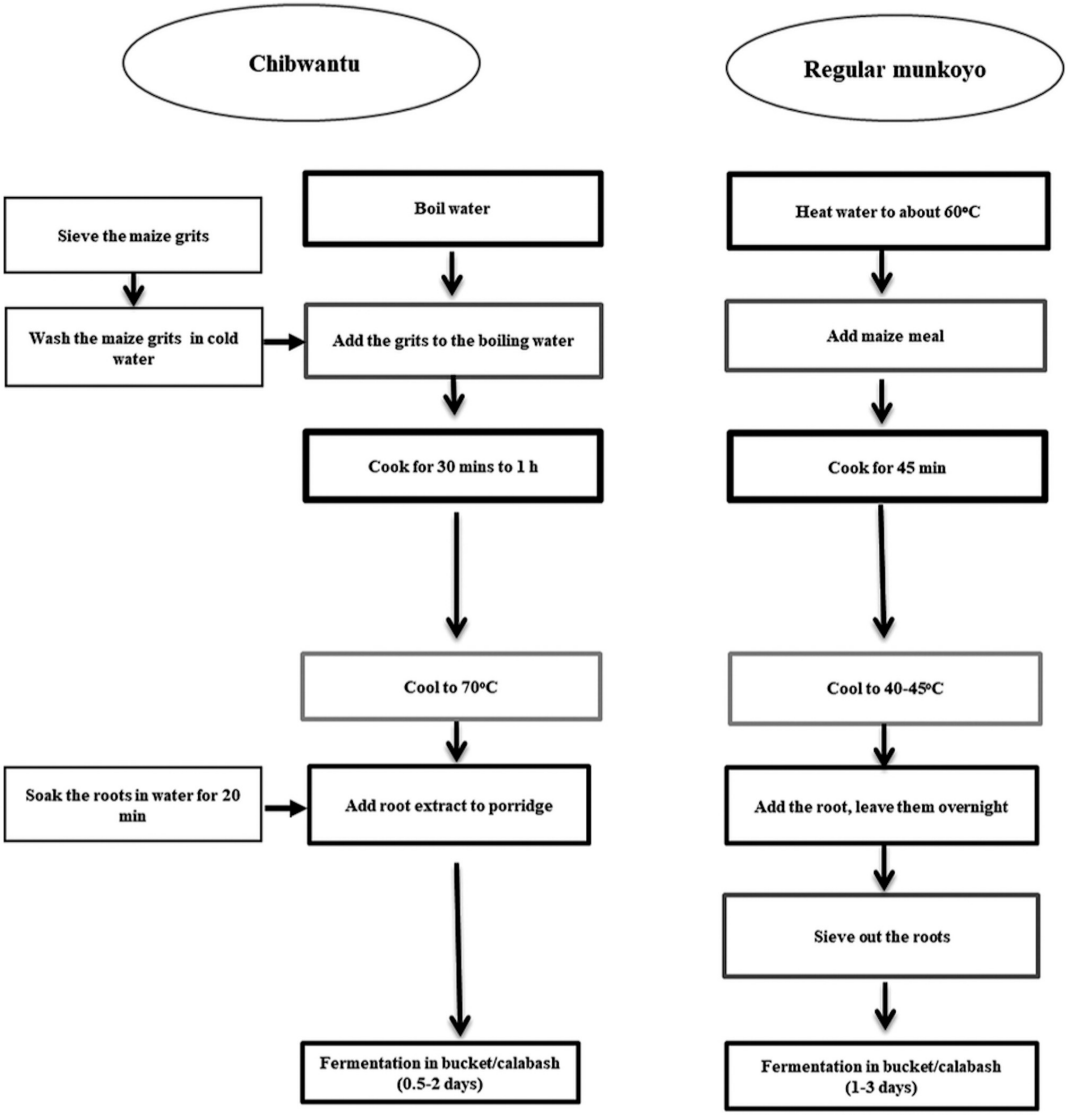
Different fermented maize beverages reported by the participants [40].

**Fig 5:**
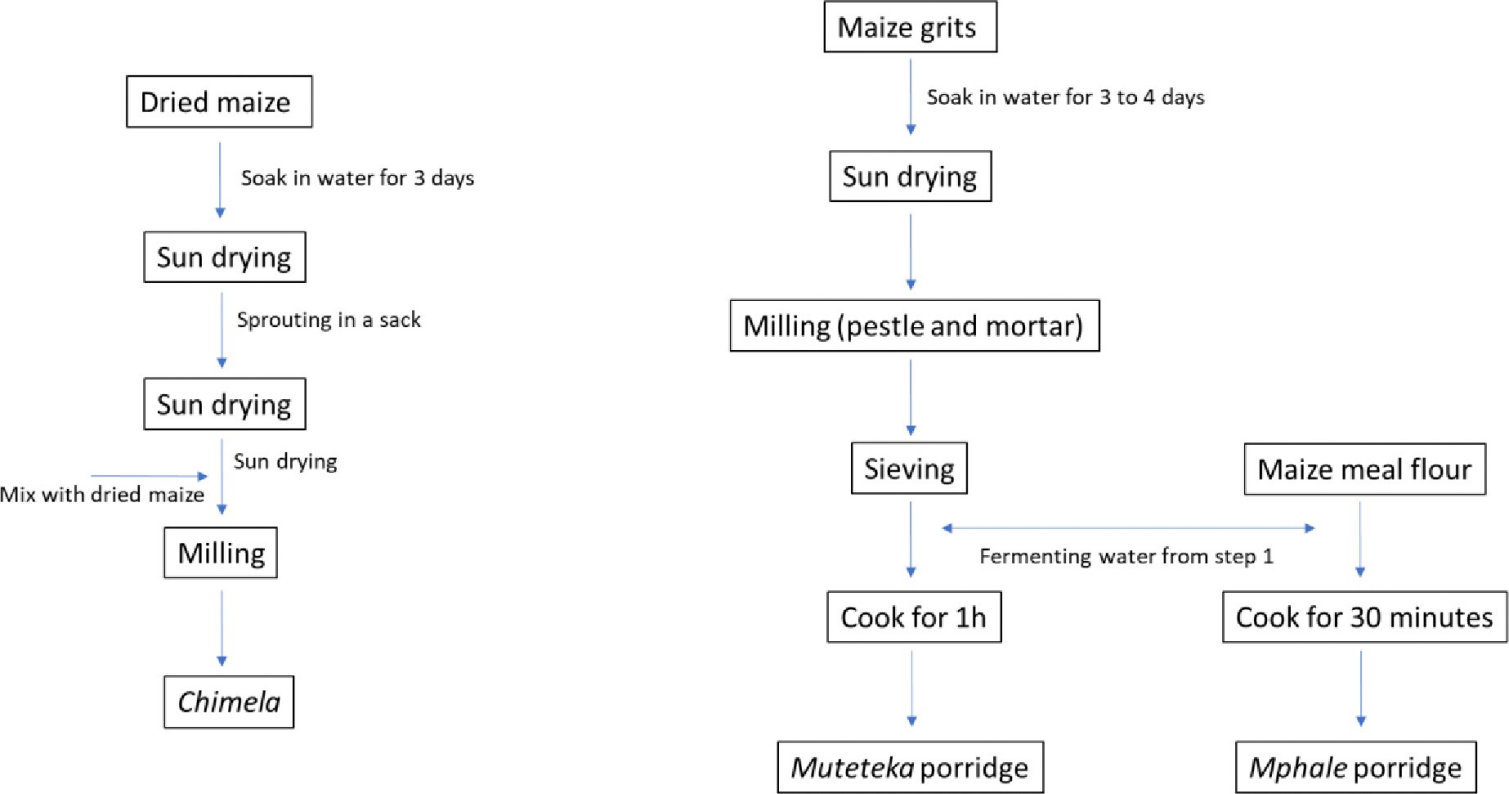
Fermented products described by participants in Eastern Province.

Besides *mabisi*, other fermented foods that were specific to different populations were produced and consumed. *Chibwantu* is a cereal-based beverage made from maize grits, with *munkoyo* roots added to speed up fermentation (Fig 4). This was typically reported by participants from Southern Province. The *munkoyo* roots are also used to produce *munkoyo*, while *tobwa* which was specific only to the Eastern Province, was reported as a commonly consumed beverage (Fig 4). While beer was also often mentioned as a common fermented beverage besides *mabisi*, some participants from Eastern Province mentioned *chimela*, *muteteka* and *mphale*. *Chimela* was used to produce *tobwa* which is a variant of munkoyo. Dried maize was soaked in water for three days and then slightly dried in the sun. It was then allowed to sprout in a sack that was placed inside the house, after which the sprouted maize was again dried in the sun before milling. Regularly dried maize was then mixed with the sprouted maize and milled together to produce *chimela*. Participants in Eastern Province also mentioned another fermented food called *muteteka* which was produced from maize grits. The grits were soaked for three to four days after which they were dried, milled using a mortar and pestle, and sieved to remove the bigger particles. The milled grits were then used to make porridge using the fermenting water and this product was called *muteteka*. They also made porridge using normally milled maize and fermenting water (from maize grits) to produce a porridge called *mphale* (Fig 5). While these foods were common during their childhood, most participants reported that they were no longer commonly produced as they now had hammermills where they could take their maize for milling rather than before when they had to use a mortar and pestle. A change in the foodscape that introduced more efficient ways of producing food resulted in a reduction in the consumption of these foods. Nonetheless, participants reported that when these foods were made available, they would consume them as a means to recollect their childhood memories and establish a connection between their children and their ancestral histories. Others mentioned that they still produced these fermented porridges as their traditional backgrounds strongly influenced their taste preferences.

#### Barriers to the consumption of healthy foods

Consumption of healthy diets can be influenced by beliefs, norms and practices. While we explored the foodscape of *mabisi* in the different subject areas, we also wanted to determine the drivers of consumption of other healthy foods and the dietary practices that acted as contributors or barriers to their consumption. When asked whether there were any foods that were not permitted in their homes during their upbringing, several participants listed foods such as fish, eggs, mice, caterpillars, and kidneys. Most participants in all three provinces reported that as children, they were not allowed to consume eggs. It was believed that consuming eggs, particularly for girls, would negatively affect their fertility and their ability to bear children, while others were told that they would lose their hair. Pregnant women were also not allowed to consume eggs as it was believed that they would give birth to children without hair, while others believed that the egg would close off the birth canal and make childbirth difficult. It was also believed in the Eastern Province that children who consumed eggs would become epileptic or they would develop ringworms. In some sectors, eggs were used for traditional purposes by witchdoctors.

Fish was also discouraged for children as it was believed to affect fertility. Other foods such as goat meat, caterpillars, and certain types of mice in Eastern Province were also forbidden for children as they were believed to cause hair loss, ringworms and rash, and infertility in girls. While these practices were followed during their upbringing, participants reported that these beliefs were no longer practiced and they believed that their parents and forefathers followed this lifestyle as a way to ration food in the house, always ensuring that the head of the household (father) had enough food to eat.

## Discussion

The foodscape has been used to describe the relationship that people have with food, their environment and their culture [10, 11]. In this paper, we employed this definition to elucidate the relationship that *mabisi* consumers and non-consumers have with *mabisi* and understand how this relationship is influenced by their environments and their culture. While *mabisi* is considered a healthy food with several health benefits, it cannot be consumed in isolation and must be consumed as part of a healthy diet. We, therefore, went further and explored the relationship that the participants had with other healthy foods and how their environment and cultural practices influenced their consumption.

Ethnicity plays an important role in both the lifestyle choices and dietary preferences of individuals. The art of *mabisi* production and consumption may be traced back to where specific ethnic groups migrated from. While several participants were unable to trace their origins, some participants from Southern Province traced their origins to northeast Africa, around the Sudanese region. The people of the Sudanese region have been linked to cattle and milk production since ancient times, with milk being an integral part of their diets, unlike other African countries [36]. Different types of fermented milk that are similar to *mabisi* are produced in Sudan. Several participants in the current study mentioned that their ancestors did not move with cattle but rather, they encountered their cattle where they settled, leading to the accidental discovery of *mabisi* production. If tracing their origins to the Sudanese region is accurate, this may suggest their preference for *mabisi*.

Despite some participants tracing their origins to northeast Africa, other participants from Southern Province were unable to trace their origins beyond their borders. The Tonga people are reported as the first Bantu-speaking group in Zambia [41]. Their traditions were reported to have replaced the iron age cultures in the area, as far back as the fifth century AD [42]. These people had a way of life that used iron and were hunters and gatherers, cultivated crops, and herded livestock such as cattle. However, Brelsford [41] suggested that these first settlers were not precisely the Tonga people but were likely the same as those identified as the “Kalomo culture” people who were later assimilated by the Tonga people migrating from the eastern side of Lake Nyasa (now called Lake Malawi) around the 15^th^ and 16^th^ centuries. Therefore, the culture of *mabisi* production and consumption may have been influenced by the assimilation and acculturation processes of both groups, which shared a history of cattle rearing.

Participants from Western Province traced their origins to the Congo region. They reported that these migrants did not move with cattle but found the cattle where they settled. When the Kololo people fleeing the Mfecane wars in South Africa in the 1800s arrived in the Barotse area, they defeated the Lozi king and ruled the Lozi people [43]. Most of the Lozi traditions were lost as a result and when the Lozi people later regained power after defeating the Lozi king, they continued with the Kololo traditions and also adopted the Kololo language [43]. This could potentially explain why their practice of *mabisi* consumption and the methods employed in its production are very similar to those produced by the people of South Africa [37, 38]. The people of South Africa used vessels such as calabashes, clay pots, and milk sacks to ferment their milk, with some of the local names being similar to those used by the Lozi people [27, 37, 38].

The Eastern Province had mixed reports for *mabisi* consumption, with most participants reporting that they were non-consumers and did not identify with it. They traced their origins to Malawi, Mozambique, and South Africa. History shows that the Nguni and Maseko that both eventually became the Ngoni people migrated north from South Africa under the leadership of Zwangendaba [43]. The Nguni Ngonis migrated through Zimbabwe into eastern Zambia (Chipata area), while the Maseko Ngonis migrated through Mozambique, into Malawi, and then further north to Tanzania [43]. During their migrations, the constant intermarriages and assimilation of those that they conquered along the way resulted in a drastic loss of the Nguni culture [41]. For example, Sinda District in Eastern Province was largely comprised of participants who were of the Nsenga tribe. Most of these participants traced their origins to Mozambique and did not identify with *mabisi* production and consumption. When the Maseko Ngonis invaded the Mozambiquan region, they found the Nsenga people with whom they intermarried and assimilated, while some fled to Zambia [41]. Likely, these people were not *mabisi* consumers and therefore the practice was never adopted.

In Malawi, the Nguni Ngonis assimilated themselves among the Chewa and Tumbuka that they found there and adopted their cultures [44]. The Tumbukas, Chewas, and Ngonis migrated into eastern Zambia following succession disputes and in search of land to cultivate. During the assimilation and acculturation process, it seems that the Nguni Ngonis may have lost their culture of *mabisi* production which is reported to have been a part of the South African people and was passed on from generation to generation [37]. While the Tumbukas and Chewas that were found in Malawi have been reported to have originally migrated from East Africa (Uganda, Tanzania) where they kept livestock such as cattle which they migrated with, it is expected that these people should have also adopted the art of *mabisi* production and consumption [41]. However, this was not the case for most of the participants who reported that their forefathers consumed a lot of milk (*chithubi*) and when it was left overnight, it was discarded. This is a practice that has continued for those that still have enough cattle for milk production. Nonetheless, based on the mixed reports, we continue to speculate that some sections of these communities may have stumbled on *mabisi* production but it may not have been widely shared in their communities and therefore not adopted as a way of life.

While several non-consumers identified *mabisi* as rotten milk that must be discarded, others acquired the taste after interactions with consumers in different parts of the country. Individuals learn what to eat and how to eat different foods through social interactions with peers, neighbors, school, and family members [45]. These play a significant role in the acculturation process when individuals are exposed to new environments and diets. However, whether or not this process becomes permanent even when individuals migrate back to their origins still needs to be explored. Our study suggests that this may potentially occur if foods such as *mabisi* and wild roots and vegetables that were no longer accessible to the participants were reintroduced and made available to them. The Kockturk-Runefors model [46] suggests that different foods are adopted based on their importance in diets. Staple foods are considered important and unlikely to change during the acculturation process, while complimentary foods may change but at a very slow process. Conversely, accessory foods are easily adopted in other cultures as they are less important in diets [28, 46]. *Mabisi* may be considered a complementary or accessory food as it is usually consumed with a staple or as an addition to the main diet. This may explain its importance in the diets of some participants that had adopted *mabisi* consumption when they interacted with consuming populations. When they returned to their original locations, they did not continue this practice. Some of the reasons reported for discontinuing *mabisi* consumption included its non-availability, high prices of commercially produced *mabisi*, little to no milk production, and losing interest in the product.

While *mabisi* provides essential nutrients such as protein and calcium to diets, diet quality which includes diversity, safety, adequacy, and moderation is important for achieving a healthy diet. Main staples such as maize, millet, sorghum, sweet potatoes, cassava; and some wild fruits, vegetables, and roots remained consistent in all three subject populations. However, complementary and accessory foods such as *mabisi*, field mice, and monitor lizards were specific to certain subject areas. These all contributed to variations in diets in each subject region. Also, cultural taboos played a role in shaping their diets. The taboo of consuming eggs by either women, children, or pregnant women was consistent in all three subject populations, also reported by Chakona & Shackleton [47]. While most of these cultural practices have been abolished, it is important to note that these may negatively affect diet quality for those that continue to practice them [47–49].

The shift from consuming traditional foods like wild animals, fish, wild fruits, and vegetables to more convenient and highly processed foods as observed by Popkin [50] in populations worldwide, was also evident in our research findings. This nutrition transition has been linked with a rise in non-communicable diseases (NCDs) which is evident in the Zambian population where the levels of obesity in women increased from 23% in 2014 to 32.5% in 2017 [51, 52]. Moreover, the methods employed to prepare food have also changed. The use of calabashes for producing *mabisi* is one such practice. Following and adopting Western lifestyles was a common response given by many participants. They chose to use plastic containers for *mabisi* production which they mentioned to be easier to use and readily available even though they admitted that *mabisi* produced in calabashes had a better taste and flavor.

Our findings provide insights into the foodscape that influences *mabisi* production and consumption and this knowledge can be used increase its consumption in areas where it is not consumed. The Zambian diet is largely made of staples and vegetables, with complementary and accessory foods differing based on the location. *Mabisi* is a rich source of nutrients such as protein, calcium, and B-vitamins which may contribute to improving the diets of the local people [17]. Further, the presence of probiotics and short-chain fatty acids (SCFAs) in *mabisi* could improve diets as these properties have been shown to prevent NCDs and dysbiosis [17, 18, 22, 26, 53]. *Mabisi* is generally inexpensive and accessible by consumers, with large quantities of milk being wasted in areas such as Southern Province where milk production is high [25]. If *mabisi* production is increased due to greater demand, this could potentially reduce milk wastage and also increase income for the local women that produce and sell it [30]. Previous studies have shown that *mabisi* production primarily occurs in rural areas, where 80% of households produce it for their own consumption, and 20% sell the product at local markets and by the roadside [27, 54].

### Strengths and limitations of the study

To our knowledge, this is the first study that has demonstrated the changing foodscape in three regions in Zambia. Many have speculated that some ethnic groups are not associated with *mabisi* consumption, but this study offers insights into the reasons behind such perceptions. Further, the study included both the elderly and the young to ensure corroboration in their narratives, recognizing that information is passed down through generations.

Our initial aim was to include more participants who were over the age of eighty as we assumed that they would have more knowledge about their origins. Unfortunately, many were unwilling to participate or unavailable during the time of the study. Another limitation of the study was that our original objective was to determine the historical perspectives of the subject population regarding *mabisi* production and consumption. However, from the responses provided, it was realized that the paper was more aligned with a foodscape perspective. If this was identified at the onset, more questions tailored towards the foodscape would have been generated. Nevertheless, the past and present foodscapes of *mabisi* production and consumption have been outlined.

## Conclusion

Understanding the foodscape that outlines the relationship that individuals have with culture, food and the environment, can be used as an entry point to boost the consumption of traditional foods such as *mabisi*. Our study shows that the changing foodscape over the years has affected the availability of different foods, while accessibility also played an important role in contributing to what was consumed. As individuals experience acculturation and assimilation in the changing foodscapes, it is possible for many to adopt the practice of *mabisi* production and consumption if the raw materials are made available. By increasing awareness and making it more available within the foodscape of non-consuming populations, *mabisi* production and consumption may increase and subsequently contribute to improving diets and overall health when consumed as part of a healthy diet. This approach also has broader implications for food security, as integrating traditional foods like *mabisi* into modern diets can help preserve cultural heritage, enhance diet quality, and strengthen local food systems. Additionally, women producers could benefit economically, improving food security for their households by generating sufficient income to feed their families.

## Supporting information

S1 Fig *Mabisi* consumption trends in Eastern Province (n = 49). Several respondents reported that *mabisi* was a culture practiced by their forefathers but discontinued through generations mainly as a result of loss of cattle to diseases.
(TIF)

S1 Table Percent distribution of ethnic origins in Southern and Western Provinces. (DOCX)

S2 Table Percent distribution of ethnic orig1ins in Eastern Province and their native custom of *mabisi* consumption. (DOCX)

S3 Table Raw socio-demographic data. (XLSX)

S1 File Questionnaire. (DOCX)

## References

[1] Zeunert J, Waterman T. Routledge handbook of landscape and food. Routledge; 2018.

[2] Fontefrancesco MF, Zocchi DM, Pieroni A. The Intersections between Food and Cultural Landscape: Insights from Three Mountain Case Studies. Land. 2023;12(3):676.

[3] Mikkelsen BE. Images of foodscapes: Introduction to foodscape studies and their application in the study of healthy eating out-of-home environments. Perspect Public Health. 2011;131(5):209–16. doi: 10.1177/1757913911415150 21999025

[4] Barnard A, Spencer J. Encyclopedia of social and cultural anthropology. Routledge; 2002.

[5] Tylor EB. Primitive culture: Researches into the development of mythology, philosophy, religion, art and custom. Vol. 2. J. Murray; 1871.

[6] Origins Krajangchom S. and localization of Tai Lue food culture in Northern Thailand. J Ethn Foods. 2023;10(1):1–11.

[7] Lavoie D, Chamlee-Wright E. Culture and enterprise: The development, representation and morality of business. Vol. 26. Psychology Press; 2000.

[8] Adema P. Garlic capital of the world: Gilroy, garlic, and the making of a festive foodscape. Univ. Press of Mississippi; 2010.

[9] HLPE. Nutrition and food systems. A report by the High Level Panel of Experts on Food Security and Nutrition of the Committee on World Food Security. Rome; 2017. Available from: http://www.fao.org/3/a-i7846e.pdf

[10] Vonthron S, Perrin C, Soulard CT. Foodscape: A scoping review and a research agenda for food security-related studies. PloS One. 2020;15(5):e0233218. doi: 10.1371/journal.pone.0233218 32433690 PMC7239489

[11] Zhu D, Wang J, Wang P, Xu H. How to frame destination foodscapes? a perspective of mixed food experience. Foods. 2022;11(12):1706. doi: 10.3390/foods11121706 35741903 PMC9222725

[12] Ingold T. The temporality of the landscape. World Archaeol. 1993;25(2):152–74.

[13] Lowitt KN. A coastal foodscape: examining the relationship between changing fisheries and community food security on the west coast of Newfoundland. Ecol Soc. 2014;19(3).

[14] Neupane S, Chimhundu R, Chan K. Cultural values affect functional food perception. Br Food J. 2019;121(8):1700–14.

[15] Tiu Wright L, Nancarrow C, Kwok PM. Food taste preferences and cultural influences on consumption. Br Food J. 2001;103(5):348–57.

[16] Chileshe J, Talsma EF, Schoustra SE, Borgonjen-Van den Berg KJ, Handema R, Zwaan BJ, et al. Potential contribution of cereal and milk based fermented foods to dietary nutrient intake of 1–5 years old children in Central province in Zambia. Plos One. 2020;15(5):e0232824. doi: 10.1371/journal.pone.0232824 32384114 PMC7209124

[17] Chileshe J, van den Heuvel J, Handema R, Zwaan BJ, Talsma EF, Schoustra S. Nutritional composition and microbial communities of two non-alcoholic traditional fermented beverages from Zambia: A study of mabisi and munkoyo. Nutrients. 2020;12(6):1628. doi: 10.3390/nu12061628 32492891 PMC7352844

[18] Moonga HB, Schoustra SE, Van den Heuvel J, Linnemann AR, Samad MS, Shindano J, et al. Composition and diversity of natural bacterial communities in mabisi, a traditionally fermented milk. Front Microbiol. 2020;1816. doi: 10.3389/fmicb.2020.01816 32849423 PMC7406715

[19] Ministry of Agriculture for Zambia, FAO. Zambia Food-Based Dietary Guidelines. Technical Recommendations 2021. Rome, Italy. Lusaka, Zambia.; 2021.

[20] D’Aimmo MR, Modesto M, Biavati B. Antibiotic resistance of lactic acid bacteria and Bifidobacterium spp. isolated from dairy and pharmaceutical products. Int J Food Microbiol. 2007;115(1):35–42. doi: 10.1016/j.ijfoodmicro.2006.10.003 17198739

[21] Egounlety M, Aworh O, Akingbala J, Houben J, Nago M. Nutritional and sensory evaluation of tempe-fortified maize-based weaning foods. Int J Food Sci Nutr. 2002;53(1):15–27. 11820093

[22] Emoto T, Yamashita T, Sasaki N, Hirota Y, Hayashi T, So A, et al. Analysis of gut microbiota in coronary artery disease patients: a possible link between gut microbiota and coronary artery disease. J Atheroscler Thromb. 2016;32672. 10.5551/jat.32672 PMC7399299 26947598

[23] Gilliland SE. Health and nutritional benefits from lactic acid bacteria. FEMS Microbiol Rev. 1990;7(1–2):175–88. doi: 10.1111/j.1574-6968.1990.tb04887.x 2271223

[24] Lourens-Hattingh A, Viljoen BC. Yogurt as probiotic carrier food. Int Dairy J. 2001;11(1–2):1–17.

[25] Moonga HB. Product optimization of Zambian traditionally fermented milk-mabisi. Wageningen University; 2019.

[26] Rogers CJ, Prabhu KS, Vijay-Kumar M. The microbiome and obesity-an established risk for certain types of cancer. Cancer J Sudbury Mass. 2014 Jun;20(3):176–80. doi: 10.1097/PPO.0000000000000049 24855004

[27] Moonga HB, Schoustra SE, Linnemann AR, Kuntashula E, Shindano J, Smid EJ. The art of mabisi production: A traditional fermented milk. PLOS ONE. 2019 Mar 14;14(3):e0213541. doi: 10.1371/journal.pone.0213541 30870441 PMC6417723

[28] Osei-Kwasi HA, Boateng D, Danquah I, Holdsworth M, Mejean C, Terragni L, et al. Acculturation and food intake among Ghanaian migrants in Europe: findings from the RODAM study. J Nutr Educ Behav. 2020;52(2):114–25. doi: 10.1016/j.jneb.2019.09.004 31601528

[29] Raza Q, Nicolaou M, Snijder MB, Stronks K, Seidell JC. Dietary acculturation among the South-Asian Surinamese population in the Netherlands: the HELIUS study. Public Health Nutr. 2017;20(11):1983–92. doi: 10.1017/S1368980016000914 27122356 PMC10261274

[30] Moonga HB, Schoustra SE, Linnemann AR, Shindano J, Smid EJ. Towards valorisation of indigenous traditional fermented milk: mabisi as a model. Curr Opin Food Sci. 2022;46:100835.

[31] Harvard University. Harvard University. Use images and media to enhance understanding. Available from: https://accessibility.huit.harvard.edu/use-images-and-media-enhance-understanding

[32] Cristancho SM, Goldszmidt M, Lingard L, Watling C. Qualitative research essentials for medical education. Singapore Med J. 2018;59(12):622. doi: 10.11622/smedj.2018093 30009321 PMC6301871

[33] Draper A, Swift JA. Qualitative research in nutrition and dietetics: Data collection issues. J Hum Nutr Diet. 2011;24(1):3–12. doi: 10.1111/j.1365-277X.2010.01117.x 21091918

[34] Schoustra SE, Kasase C, Toarta C, Kassen R, Poulain AJ. Microbial community structure of three traditional Zambian fermented products: mabisi, chibwantu and munkoyo. PLoS One. 2013;8(5). doi: 10.1371/journal.pone.0063948 23691123 PMC3653860

[35] Braun V, Clarke V. Successful qualitative research: A practical guide for beginners. sage; 2013.

[36] Abdelgadir WS, Ahmed TK, Dirar HA. The traditional fermented milk products of the Sudan. Int J Food Microbiol. 1998;44(1–2):1–13. doi: 10.1016/s0168-1605(98)00090-7 9849779

[37] Beukes EM, Bester BH, Mostert JF. The microbiology of South African traditional fermented milks. Int J Food Microbiol. 2001;63(3):189–97. doi: 10.1016/s0168-1605(00)00417-7 11246902

[38] Fox FW. Some Bantu Recipes from the Eastern Cape Province. Bantu Stud. 1939;13(1):65–74.

[39] Rigby CW, Rosen A, Berry HL, Hart CR. If the land’s sick, we’re sick:* The impact of prolonged drought on the social and emotional well‐being of Aboriginal communities in rural New South Wales. Aust J Rural Health. 2011;19(5):249–54. doi: 10.1111/j.1440-1584.2011.01223.x 21933367

[40] Moonga HB, Phiri S, Schoustra SE, Chileshe J, Chirwa-Moonga T, Shindano J. The Munkoyo Root: Traditional Uses, Biochemistry, Fermentation, and Potential Cultivation. In: African Natural Plant Products, Volume III: Discoveries and Innovations in Chemistry, Bioactivity, and Applications. American Chemical Society; 2020. p. 81–99. (ACS Symposium Series; vol. 1361). Available from: 10.1021/bk-2020-1361.ch004

[41] Brelsford WV. The tribes of Zambia. The Government Printer, Lusaka; 1965.

[42] Vickery KP. Black and White in Southern Zambia: the Tonga plateau economy and British imperialism, 1890–1939. Vol. 21. Greenwood Publishing Group; 1986.

[43] Flint JE, Flint JE, Oliver RA. The Cambridge History of Africa. Vol. 5. Cambridge University Press; 1975.

[44] Madise S. The Ngoni of Malawi (A History Revisited). Available SSRN 2587050. 2015;

[45] Monterrosa EC, Frongillo EA, Drewnowski A, de Pee S, Vandevijvere S. Sociocultural influences on food choices and implications for sustainable healthy diets. Food Nutr Bull. 2020;41(2_suppl):59S–73S. doi: 10.1177/0379572120975874 33356592

[46] Kockturk-Runefors T. A model for adaptation to a new food pattern: the case of immigrants. Appetite. 1991;16(2):163.

[47] Chakona G, Shackleton C. Food taboos and cultural beliefs influence food choice and dietary preferences among pregnant women in the Eastern Cape, South Africa. Nutrients. 2019;11(11):2668. doi: 10.3390/nu11112668 31694181 PMC6893604

[48] Ekwochi U, Osuorah CD, Ndu IK, Ifediora C, Asinobi IN, Eke CB. Food taboos and myths in South Eastern Nigeria: The belief and practice of mothers in the region. J Ethnobiol Ethnomedicine. 2016;12(1):1–6. doi: 10.1186/s13002-016-0079-x 26818243 PMC4729178

[49] Kariuki LW, Lambert C, Purwestri RC, Maundu P, Biesalski HK. Role of food taboos in energy, macro and micronutrient intake of pregnant women in western Kenya. Nutr Food Sci. 2017;

[50] Popkin BM. Urbanization, lifestyle changes and the nutrition transition. World Dev. 1999;27(11):1905–16.

[51] Central Statistical Office (CSO Zambia, Ministry of Health (MOH) Zambia, ICF International. Zambia Demographic and Health Survey 2013–2014. Rockville Maryland, USA: Central Statistical Office, Ministry of Health, and ICF International; 2014.

[52] WHO. Zambia Steps For Non Communicable Diseases Risk Factors. Ministry of Health and World Health Organization; 2017. Available from: http://www.who.int/ncds/surveillance/steps/zambia/en/

[53] Gross M. Does the gut microbiome hold clues to obesity and diabetes? Curr Biol CB. 2013 May 6;23(9):R359–362. doi: 10.1016/j.cub.2013.04.047 23802281

[54] Chileshe J. Nutrition, health and microbial ecology of traditional fermented foods in Zambia. [Wageningen]: Wageningen University; 2019.

