# Supplementary figures and images for: Past and present foodscapes of a traditional fermented milk, mabisi, in three Zambian regions
Source: PLoS One. 2024 Dec 31;19(12):e0310507. doi: 10.1371/journal.pone.0310507 (PMC11687773; doi:10.1371/journal.pone.0310507)

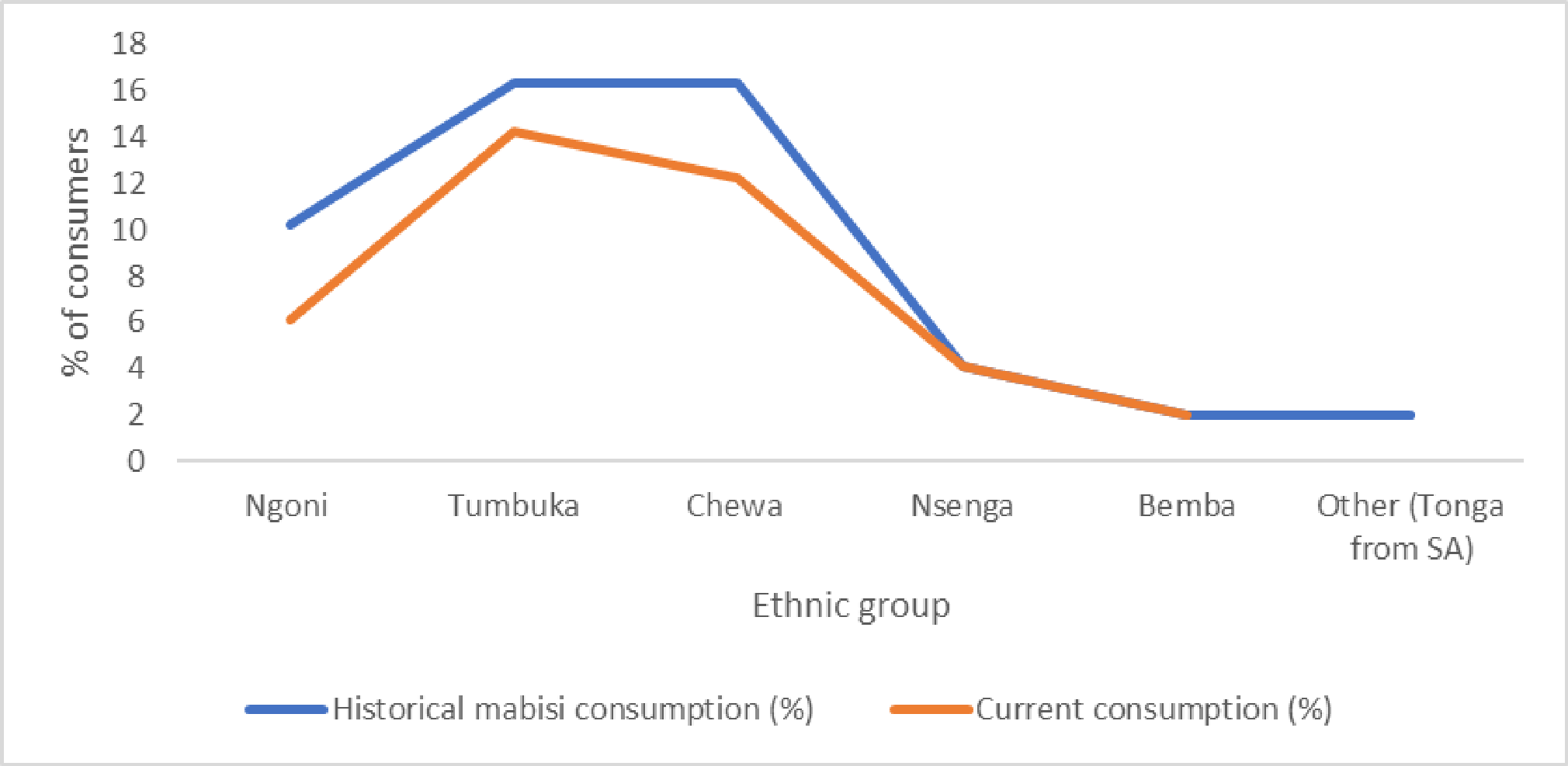

Supplement: S1 Fig — Several respondents reported that mabisi was a culture practiced by their forefathers but discontinued through generations mainly as a result of loss of cattle to diseases. (TIF) [file pone.0310507.s001.tif]
