# Supplementary material for: Past and present foodscapes of a traditional fermented milk, mabisi, in three Zambian regions
Source: PLoS One. 2024 Dec 31;19(12):e0310507. doi: 10.1371/journal.pone.0310507 (PMC11687773; doi:10.1371/journal.pone.0310507)
Supplement: S1 Table — (DOCX) [file pone.0310507.s002.docx]

| **Southern Province** | | | | | | | | | |
| --- | --- | --- | --- | --- | --- | --- | --- | --- | --- |
| **District** |  | **Reported country/ region of origin** | | | | | | | |
|  | **Ethnic group** | Zambia | Congo | South Africa | East Africa/ Sudan | Zimbabwe | Namibia | Angola | I don’t know |
| **Choma (n=18)** | Tonga | 56 |  |  |  | 11 |  |  | 11 |
|  | Ila |  |  |  | 11 |  |  |  | 6 |
|  | Lozi |  | 6 |  |  |  |  |  |  |
| **Western Province** | | | | | | | | | |
| **Mongu (n=20)** | Lozi |  | 10 | 10 | 5 |  | 10 |  | 35 |
|  | Mbunda |  | 5 |  |  |  |  | 10 |  |
|  | Tokaleya | 15 |  |  |  |  |  |  |  |
