# Supplementary material for: Past and present foodscapes of a traditional fermented milk, mabisi, in three Zambian regions
Source: PLoS One. 2024 Dec 31;19(12):e0310507. doi: 10.1371/journal.pone.0310507 (PMC11687773; doi:10.1371/journal.pone.0310507)
Supplement: S2 Table — (DOCX) [file pone.0310507.s003.docx]

| **District** |  | **Reported country/ region of origin** | | | | | | **Native *mabisi* consumption** | | |
| --- | --- | --- | --- | --- | --- | --- | --- | --- | --- | --- |
| **Chipata**  **(n = 20)** | **Ethnic group** | **Zambia** | **Congo** | **Mozambique** | **Malawi** | **South Africa** | **I don't know** | **Consumers** | **Non-consumers** | **I don't know** |
|  | Ngoni |  |  |  |  | 10 | 25 | 20 | 5 | 10 |
|  | Tumbuka |  |  |  |  | 10 | 5 | 10 |  | 5 |
|  | Other (Tonga from SA) |  |  |  |  | 5 |  | 5 |  |  |
|  | Chewa | 5 | 5 | 5 | 20 | 5 |  | 15 | 5 | 20 |
|  | Nsenga |  |  | 5 |  |  |  |  | 5 |  |
|  | Ngoni |  |  | 6 |  |  |  | 6 |  |  |
|  | Tumbuka |  |  |  | 61 |  | 6 | 33 | 28 | 6 |
| **Lundazi**  **(n = 18)** | Chewa |  |  |  | 6 |  | 11 | 11 | 6 |  |
|  | Bemba |  | 6 |  |  |  |  | 6 |  |  |
|  | Other (Kunda) |  |  |  |  |  | 6 |  | 6 |  |
| **Sinda**  **(n=12)** | Chewa |  |  | 17 |  |  | 8 | 23 |  |  |
|  | Nsenga |  |  | 58 |  |  | 17 | 15 | 46 | 15 |
