# Supplementary material for: Past and present foodscapes of a traditional fermented milk, mabisi, in three Zambian regions
Source: PLoS One. 2024 Dec 31;19(12):e0310507. doi: 10.1371/journal.pone.0310507 (PMC11687773; doi:10.1371/journal.pone.0310507)
Supplement: S1 File — (DOCX) [file pone.0310507.s005.docx]

**QUESTIONNAIRE ON THE STUDY ”HISTORICAL PERSPECTIVES AND USE OF *MABISI”***

**SECTION A: INTRODUCTION TO THE RESEARCH AND PARTICIPANT CONSENT**

1. **Greetings and Introduction**

Dear Respondent,

My name is ................... and I am a research assistant working on a study entitled, “Historical perspectives and use of *mabisi*.” The aims of the study are to determine the origins of production and consumption of *mabisi* and also determine whether any cultural influences exist in its production and consumption. We would like to know the history that influences *mabisi* consumption and production. The study will be carried out in Choma and Chipata districts. This research is part of a sandwich PhD project which is in collaboration with Wageningen University of The Netherlands and the University of Zambia, being carried out by Taonga Chirwa-Moonga. We thank you in advance for your participation.

1. **Interview process and confidentiality**

I will use the interview guide that I have with me to ask you questions on your knowledge of this topic and I will use a voice recorder to record the information you provide me with. Everything that is discussed here will be used only for research purposes and your identity will be kept confidential.

1. **Voluntary participation**

Participation is completely voluntary and should you want to discontinue or decline to answer any questions, feel free to do so. I however encourage you to participate in the study as the findings will be useful in providing baseline information for future studies.

1. **Consent**

I have read and understood the information above regarding this research study and I voluntarily

consent to participate.

Name: .......................................................................................................

Sign: .........................................................................................................

Date: ..........................................................................................................

**SECTION B**

1. **Questionnaire administration information**

| **N°** | **Questions** | **Answers** | **Code** |
| --- | --- | --- | --- |
| 1.1 | Name of enumerator |  |  |
| 1.2 | Name of respondent |  |  |
| 1.3 | Date of interview |  |  |
| 1.4 | Time | Start :  Finish : |  |
| 1.5 | Location | Village....................................................................  Camp.....................................................................  Block...................................................................  District.................................................................  Province............................................................... |  |
| 1.6 | Contact Number (Mobile phone) |  |  |
| 1.7 | Main language used by respondent | 1. English 2. Tonga 3. Ila 4. Lozi 5. Nyanja/ Nsenga 6. Ngoni 7. Chewa 8. Tumbuka 9. Bemba 10. Other (specify) ........................................................ |  |
| 1.8 | Was a translator used? | 1. Yes 2. No |  |

1. **Socio - demographic information**

| **N°** | **Questions** | **Answers** | **Code** |
| --- | --- | --- | --- |
| 2.1 | Gender | 1. Male  2. Female |  |
| 2.2 | Location | Village....................................................................  Camp.....................................................................  Block...................................................................  District.................................................................  Town....................................................................  Province............................................................... |  |
| 2.3 | Ethnic group (Tribe) | 1. Tonga 2. Ila 3. Lozi 4. Nsenga 5. Ngoni 6. Chewa 7. Tumbuka 8. Bemba 9. Other (specify) ......................................................... |  |
| 2.4 | Marital status | 1. Single  2. Married  3. Divorced  4. Widow/ widower |  |
| 2.5 | Age in years | 1. ≤18  2. 19-29  3. 30-39  4. 40-49  5. 50-59  6. 60+ |  |
| 2.6 | Education level | 1. No education  2. Primary (Grade 7)  3. Secondary (O-Level or A-Level)  4. Vocational training  5. Tertiary (diploma or degree)  6. Other (specify)......................................................................... |  |
| 2.7 | Who is head of the household? | 1. Father 2. Mother 3. Child 4. Grandmother 5. Grandfather 6. Aunt 7. Uncle 8. Other (specify)....................................................................... |  |
| 2.8 | Size of the Household | 1. 0-6 2. 6-10 3. >10 4. Other (specify) …………………………………………………………………. |  |
| 2.9 | Source of income | 1. Full time employment 2. Part-time employment 3. Agriculture 4. Business 5. Supported by family member 6. Other (specify) ....................................................................... |  |
| 2.10 | Monthly household income | 1. <K100 2. K100 – K400 3. K500 – K999 4. K1000 – K1500 5. K1501 – K2000 6. >K2000 |  |

**SECTION C – Interview guide**

1. **Participant background**
2. Could you tell me a little about yourself, where you were born and your upbringing?
3. When you were growing up, what cultural beliefs/ traditions did you have in your family that may have affected the type of food you ate? (e.g church, food taboos, etc)
4. Could you give some examples of foods that were popular in your home?
5. Was your family involved in any kind of agricultural work, including livestock production?
6. Were these foods eaten by the family (or was this strictly for sale purposes?
7. What do you currently do?
8. Do you own livestock? If yes, what types and how many?
9. For how long have you lived in this community and how has this affected the type of food you consume?
10. ***Mabisi* production and consumption**
11. Are you familiar with *mabisi?*
12. Kindly explain to me what *mabisi* is and what it is used for.
13. Kindly tell me how *mabisi* is produced.
    1. How long does it takes to produce it
14. Is *mabisi* popular in your area and how is it normally consumed here?
    1. Is *mabisi* eaten hot or cold?
15. Are there any special reasons for consuming *mabisi* that you know about?
16. Is *mabisi* from goats popular here? If not, why?
17. What other fermented foods are popular here in your area?
18. **Cultural/ traditional uses (*mabisi* consumers)**
    1. Briefly explain to me about where your ethnic group/ tribe came from and how they came to settle down in this area
    2. What kinds of food did your forefathers eat?
    3. Are these foods still consumed at present? Are they all still readily available?
    4. Was *mabisi* part of the foods that they ate? If yes, kindly explain how they first learnt of making and consuming *mabisi*?
    5. How is *mabisi* produced in your tradition?
    6. Has the production of *mabisi* ever changed with time or is this still the same?
    7. Are there any traditional/ religious/ medicinal uses/ beliefs of *mabisi*?
    8. What are your thoughts about *mabisi* in general? Do you think that it can be used to curb malnutrition and disease in your area?
    9. Is *mabisi* considered important for pregnant women? Children? Why?
